# Replication protein A: a multifunctional protein with roles in DNA replication, repair and beyond

**DOI:** 10.1093/narcan/zcaa022

**Published:** 2020-09-25

**Authors:** Rositsa Dueva, George Iliakis

**Affiliations:** Institute of Medical Radiation Biology, University of Duisburg–Essen Medical School, 45122 Essen, Germany; Institute of Physiology, University of Duisburg–Essen Medical School, 45122 Essen, Germany; Institute of Medical Radiation Biology, University of Duisburg–Essen Medical School, 45122 Essen, Germany

## Abstract

Single-stranded DNA (ssDNA) forms continuously during DNA replication and is an important intermediate during recombination-mediated repair of damaged DNA. Replication protein A (RPA) is the major eukaryotic ssDNA-binding protein. As such, RPA protects the transiently formed ssDNA from nucleolytic degradation and serves as a physical platform for the recruitment of DNA damage response factors. Prominent and well-studied RPA-interacting partners are the tumor suppressor protein p53, the RAD51 recombinase and the ATR-interacting proteins ATRIP and ETAA1. RPA interactions are also documented with the helicases BLM, WRN and SMARCAL1/HARP, as well as the nucleotide excision repair proteins XPA, XPG and XPF–ERCC1. Besides its well-studied roles in DNA replication (restart) and repair, accumulating evidence shows that RPA is engaged in DNA activities in a broader biological context, including nucleosome assembly on nascent chromatin, regulation of gene expression, telomere maintenance and numerous other aspects of nucleic acid metabolism. In addition, novel RPA inhibitors show promising effects in cancer treatment, as single agents or in combination with chemotherapeutics. Since the biochemical properties of RPA and its roles in DNA repair have been extensively reviewed, here we focus on recent discoveries describing several non-canonical functions.

## MODES OF SSDNA BINDING: RPA LOADING, DIFFUSION AND DISSOCIATION

### RPA loading and diffusion along ssDNA

**Figure 1.**
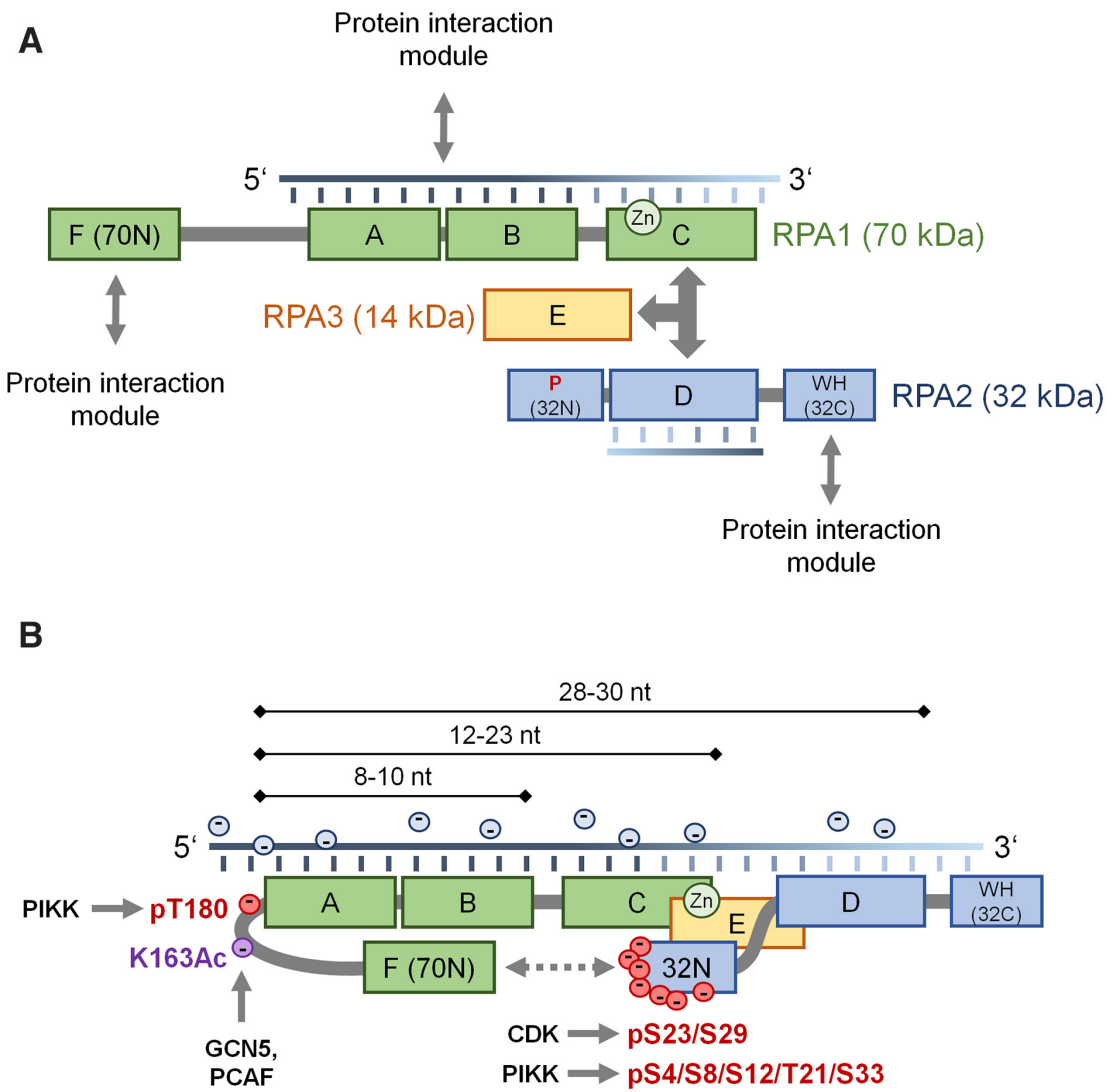
Schematic representation of RPA domains. (**A**) The domains in each subunit of RPA complex are joined by flexible linkers. RPA has four ssDNA-binding domains with DBD-A and DBD-B being high-affinity ssDNA-binding domains, as indicated by intensity gradient in ssDNA. The N-terminal domain of RPA1 (DBD-F) is involved in protein–protein interactions including tumor suppressor p53. Zinc finger motif in the C-terminal fold of 70 kDa subunit provides structural stability and has a positive role in RPA’s DNA-binding activity. The phosphorylation motif is located in the N-terminus of RPA2. RPA32C contains a winged helix–turn–helix (WH) fold involved in protein–protein interactions. Triple arrow represents the inter-subunit interactions, known as the RPA trimerization core. Two-headed arrows represent protein–protein interactions. (**B**) Final stage of RPA binding to ssDNA of around 30 nt. Upon DNA damage, RPA gains several negative charges through phosphorylation, primarily on the N-terminal domain of RPA2 (32N), which alters RPA conformation and induces its physical interaction with the N-terminus of RPA1 (128). Electrostatic repulsive forces between hyperphosphorylated RPA2 and negatively charged ssDNA may foster RPA dissociation from ssDNA.

Replication protein A (RPA), originally identified as an essential factor for SV40 DNA replication *in vitro* (1–4), is now established as an essential component of several aspects of the DNA metabolism, such as replication, repair and recombination. In eukaryotes, RPA is an abundant multifunctional single-stranded DNA (ssDNA)-binding protein complex consisting of three tightly associated subunits (70, 34 and 14 kDa), named RPA1, RPA2 and RPA3, with order determined by molecular weight. The RPA complex contains six oligonucleotide/oligosaccharide-binding (OB)-fold domains that assume an architecture common to several ssDNA-binding proteins (SSBs). Four of these OB folds, also termed DNA-binding domains (DBDs), DBD-A, DBD-B, DBD-C and DBD-F, are located in the largest RPA1 subunit. DBD-D resides on the mid-sized RPA2, while DBD-E is situated in the smallest RPA3 subunit. It is thought that DBD-C, DBD-D and DBD-E mediate inter-subunit interactions (trimerization core), while DBD-A, DBD-B, DBD-C and DBD-D are involved in ssDNA binding, with DBD-A and DBD-B dominating this interaction (5,6) (Figure 1A). However, a direct interaction between RPA3 and ssDNA was also reported (7). The zinc finger motif in DBD-C provides structural stability and enhances RPA’s DNA-binding activity (8–12). The protein interaction modules of RPA are located in the N-terminal domain of RPA1 (70N), which harbors DBD-F, as well as in the C-terminus of RPA2 (32C), while the N-terminus of RPA2 is the primary phosphorylation site of the protein (Figure 1A).

RPA binds to ssDNA in a sequence-independent manner with a dissociation constant *K*_D_ of ∼10^−7^ to 10^−10^ M (13) and a 5′ → 3′ polarity, where the strong ssDNA interaction domain DBD-A binds to the 5′ end of the ssDNA, followed by DBD-B, while the weak ssDNA-binding domains DBD-C and DBD-D are positioned toward the 3′ side (Figure 1B) (14–17). Despite this high affinity, the RPA–ssDNA complex is not static. Extensive research has revealed that the six DBDs can adopt multiple conformations, making RPA extremely flexible and able to bind ssDNA in modes that depend on ssDNA length and the participating DBDs (Figure 1B) (18–26) [reviewed in (17,27,28)]. Indeed, RPA bound to ssDNA is rapidly exchanged when free RPA or other ssDNA-binding proteins, such as RAD51, are present (29,30). A specific mutation in the large subunit of yeast RPA1 (K45E) affects RPA displacement by RAD51 (31), while biochemical studies indicate that RPA undergoes sliding diffusion along ssDNA that melts hairpin structures (32,33). Recently, it was proposed that transient interactions occurring during sliding diffusion of yeast RPA on DNA involve DBD-A of RPA1 and DBD-E of RPA3 (34). The model is that phosphorylation of RPA1 at S178 enhances the DBD-A–DBD-E interaction and the cooperative behavior of RPA on ssDNA; all this facilitates displacement of RPA from ssDNA and allows access to factors such as RAD51. The study establishes that RPA3 and its DBD-E domain are essential components of the functional RPA–ssDNA complex (34). The migration of long linear polymers in a concentrated and entangled system, such as DNA in the cell nucleus, can be achieved by a process known as ‘reptation’ (35). This concept is comparable to the wavy motion of snakes. Recent findings further substantiate the concept that ssDNA diffuses along RPA, and *Escherichia coli* SSB is indeed utilizing a reptation mechanism (36–38). This diffusion mechanism involves the migration of small stretches of ssDNA (1–7 nt), stored in transient bulges. The bulge formation is facilitated by the short-range interactions between the bases of ssDNA and the aromatic side chains of RPA. The boundaries of these bulging segments are defined by the points at which a few contacts between ssDNA and the RPA interface are broken. Long-range electrostatic interactions between positively charged amino acid residues of RPA and the ssDNA phosphate groups enable the release of the stored ssDNA in the bulge. In this way, despite the extensive ssDNA–RPA interactions, the bulge formation enables a stepwise diffusion of ssDNA along its RPA-binding interface (36).

Although RPA has a high affinity for ssDNA *in vitro*, its loading on ssDNA in the complex cellular environment may rely on additional cofactor(s). A recent study describes how RPA is loaded on ssDNA regions in budding yeast (39). It was also demonstrated that RPA loading on ssDNA is also assisted by Cdc45, an essential component of the replicative DNA helicase (40).

### Nuclear import of RPA

It is been proposed that yeast regulator of Ty1 transposition 105 (Rtt105) acts as a chaperone for RPA. Rtt105 directly binds to RPA during S phase, and together with importin β (Kap95 in yeast) (41) mediates RPA’s nuclear import. Moreover, Rtt105 promotes RPA loading on ssDNA at both active and HU-stalled replication forks without being present at the final RPA–ssDNA complex (39). Furthermore, an SSB encoded by Rim1 that is essential for mitochondrial DNA replication in yeast also co-purifies with Rtt105. This function of Rtt105 is reminiscent of that of histone chaperones, which are responsible for the nuclear import of histones, the major double-stranded DNA (dsDNA)-binding proteins in eukaryotic cells, and thus for the formation of nucleosomes. However, Rtt105 orthologs have not been found in higher eukaryotes. The authors propose that XRIPα, an RPA-binding protein that is required for RPA’s nuclear import in *Xenopus* (42), could be a functional homolog of Rtt105 in higher eukaryotes (39).

### RPA dissociation from ssDNA

The dissociation of RPA from ssDNA remains speculative. It was proposed that the DBDs dissociate from ssDNA in reverse order (from 3′ to 5′ end). Binding of other proteins may also change RPA conformation to a compact, weaker binding mode, thus enabling its dissociation (27). The most prominent example is RPA displacement by RAD51 recombinase (43,44). The list of DNA processing proteins that interact with RPA and probably remodel its DNA-binding mode is growing constantly (Table 1).

**Table 1.** RPA-interacting partners

RPA-interacting partner	Supporting function	References
53BP1	DNA repair	(45)
AID (activation-induced cytidine deaminase)	Immunoglobulin diversification	(46)
Ajuba	DNA damage response (DDR)	(47,48)
ATRIP	Checkpoint signaling, DNA repair	(49–51)
BID	Replication stress response	(52)
BLM	DNA unwinding, resection	(53,54)
BRCA2	Recombination	(55)
Cdc13, *Saccharomyces cerevisiae*	Telomere maintenance	(56)
Cdc45	RPA loading on ssDNA	(40)
DNA2	Recombination	(57,58)
DNA-PKcs	DNA repair	(59,60)
DSS1	Recombination	(61)
ETTA1	ATR activation, repair at stalled replication forks	(62–65)
FACT	Chromatin remodeling	(66,67)
FANCJ	DNA repair, genome stability	(68)
HERC2	Replication	(69,70)
HIRA	Chromatin remodeling	(71)
Histones H3 and H4	Chromatin remodeling	(72)
HSF1	Gene expression	(66)
KU, *S. cerevisiae* and *S. pombe*	Telomere maintenance, end resection	(56,73)
Menin	Genome stability	(74,75)
MRE11–RAD50–NBS1	DNA end resection	(76,77)
Nucleolin	Replication (stress)	(78–80)
p53	DNA double-strand break (DSB) repair by homologous recombination (HR)	(81–86)
PALB2	Recovery of stalled replication forks	(87)
PrimPol	Replication restart, DNA damage tolerance	(88–90)
PRP19	DNA repair	(91)
PTEN	Genome stability	(92)
Rad9/Rad1/Hus1 (9-1-1)	DDR	(93,94)
Rad18	Monoubiquitylation of proliferating cell nuclear antigen (PCNA) during replication	(95,96)
RAD51	Recombination	(43,44,97,98)
Rad52, human	DNA repair	(97–99)
Rad52, *S. cerevisiae*	Recombination	(100–103)
RFWD3	DNA repair	(104,105)
RNaseH	Transcription, DNA repair	(106)
Rtt105, *S. cerevisiae*	Acts as a chaperone for RPA	(39)
SENP6	Unperturbed DNA replication	(107)
SMARCAL1/HARP	Replication fork restart	(108–111)
Tipin (Timeless-interacting protein)	DDR	(112,113)
UNG2	Base excision repair	(114,115)
WRN	DNA unwinding, resection	(54,116–119)
XPA	Nucleotide excision repair (NER)	(114,120–124)
XPG	NER	(14,125)
XPF–ERCC1	NER	(14,125–127)
XRIPα, *Xenopus laevis*	Nuclear import of RPA	(42)

Electrostatic repulsive forces can also add up to RPA unloading. Post-translational modifications of RPA providing a massive negative charge, such as phosphorylation and acetylation, may loosen the interaction between RPA and the negatively charged ssDNA (Figure 1B) (128).

## RPA AS A REPLISOME COMPONENT

### RPA in DNA replication

**Figure 2.**
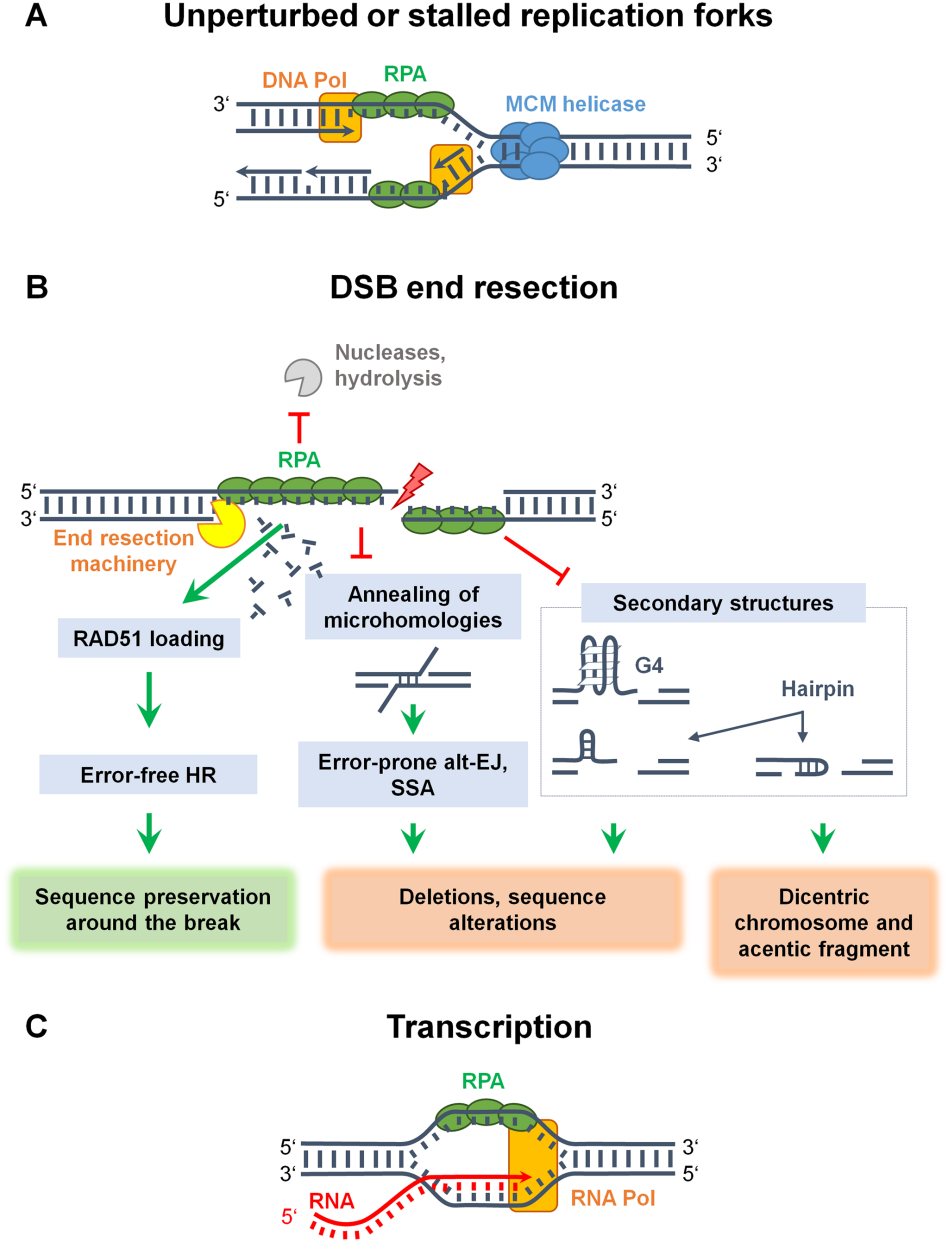
RPA binding to ssDNA intermediates. (**A**) RPA binds to ssDNA intermediates during DNA synthesis under normal conditions and when replication forks are stalled by genotoxic agents. (**B**) DNA end resection also creates ssDNA, which is substrate for all forms of homology-directed repair (HDR). RPA has a protective role against nucleases and formation of secondary structures such as G-quadruplexes (G4s) and hairpins, thus supporting RAD51-mediated HR. Furthermore, RPA prevents spontaneous annealing of microhomologies and inverted repeats that can lead to deletions, sequence alterations or chromosome breakage with the accompanying dicentric chromosomes and acentric fragments. (**C**) The displaced ssDNA strand during transcription can be recognized by RPA in the absence of DNA damage. Note that other components involved in these processes are not shown for simplicity.

RPA is essential for DNA replication and cell cycle progression, as it protects the transiently formed ssDNA from nucleolytic degradation and secondary structure formation, but its necessity for replication goes beyond this protective function (Figure 2A). DNA-dependent DNA polymerases synthesize new DNA strands using deoxyribonucleotides with a high degree of accuracy and efficiency, and RPA stimulates the activity of DNA Pol α and Pol δ (129,130). Polymerases add nucleotides only onto a pre-existing 3′-OH end and therefore require DNA primases that synthesize short RNA segments, called primers, to initiate DNA replication. Human primase–polymerase (hPrimPol1) was identified as a novel interacting partner of RPA, with the interaction mediated by RPA1’s N-terminal domain. Human PrimPol belongs to the archaeo-eukaryotic primase superfamily and displays both primase and DNA damage tolerance polymerase activities. Furthermore, the hPrimPol–RPA interaction is important for the restoration of DNA synthesis following replication fork stalling (88). Other reports confirm the interaction between PrimPol and RPA1 and demonstrate that PrimPol also interacts with the mitochondrial SSB (mtSSB). Surprisingly, however, both RPA and mtSSB severely suppress primer synthesis and extension by PrimPol *in vitro*, probably by blocking PrimPol-binding sites on ssDNA. Mutagenesis assays also reveal that PrimPol is highly error-prone, generating insertion–deletion errors, explaining the requirement for its tight regulation during DNA synthesis. Collectively, these observations led to the assumption that RPA and mtSSB restrict the polymerase activity of PrimPol at stalled replication forks to suppress mutagenesis (89). Studies on the molecular basis of RPA–PrimPol interaction during repriming revealed that PrimPol has two RPA-interacting motifs (termed RBM-A and RBM-B) in its C-terminal domain, binding to the basic cleft of DBD-F. RBM-A has a primary role in mediating RPA–PrimPol interaction *in vivo* (90). Despite reports on RPA inhibiting PrimPol to suppress mutagenesis (89), biochemical analyses reveal that RPA also elicits stimulatory effects on both primase and polymerase activities of PrimPol, but specifically on long ssDNA templates (90,131). Thus, there seems to be considerable plasticity in the interactions between RPA and PrimPol and their ultimate effects on DNA replication.

The DNA damage tolerance pathways, where PrimPol is involved, permit lesion bypass during DNA synthesis that can be carried through translesion synthesis (132,133). Thereby, the sliding clamp PCNA serves as a polymerase processivity factor. Several studies implicate RPA in DNA damage tolerance, where it regulates the DNA damage-induced mono-ubiquitylation of PCNA (95,96,134,135). RPA interacts with Rad18, the ubiquitin ligase responsible for PCNA mono-ubiquitylation, which likely drives Rad18 recruitment to ssDNA (95,96,135). Other reports suggest that RPA alone regulates PCNA sliding along ssDNA within post-replicative gaps (136).

RPA is also implicated in histone deposition during DNA replication through a direct interaction between the N-terminus of RPA1 and the Pob3 subunit of the yeast histone chaperone complex FACT (67). A novel and intriguing study suggests that RPA, together with specific histone H3–H4 chaperones, acts in replication-coupled nucleosome assembly. While residing on ssDNA, RPA may directly bind free H3–H4 complexes and deposit them onto adjacent newly replicated dsDNA (72). Hence, RPA is multitasking at the replication fork—safeguarding DNA integrity during replication and facilitating the formation of new chromatin.

### RPA in checkpoint signaling

DNA synthesis is a highly regulated process to guarantee precise duplication of the genome. Slowing or stalling of replication fork progression by various endogenous and exogenous stresses can endanger the integrity of DNA replication. High levels of replication stress usually lead to DNA damage and threaten genomic stability (137). Template switching during replication fork repair necessitates realignment of the nascent ssDNA strand to initiate DNA synthesis from an alternative template. RPA-coated ssDNA regions at stalled forks trigger ATR recruitment, which phosphorylates several downstream targets, including the CHK1 effector kinase and the tumor suppressor p53. Thus, ATR signaling delays cell cycle progression and ensures replication fork stabilization (138). A critical regulatory partner of ATR is ATR-interacting protein (ATRIP), which localizes ATR to DNA damage sites or stalled replication forks through an interaction with RPA-coated ssDNA (49–51,139). Until recently, TopBP1 was considered as the only activator of ATR–ATRIP complex in vertebrates (140). However, it is now evident that the Ewing’s tumor-associated antigen 1 (ETAA1) avidly interacts with RPA, to localize at stalled replication forks and activate ATR (62–65). ETAA1 recruitment to stalled replication forks depends on its interaction with two RPA domains—70N and 32C. Because ETAA1-deficient cells exhibit defective RPA2 phosphorylation, ETAA1 may facilitate the proper phosphorylation of RPA2 (63–65). Notably, when RPA is downregulated, other SSBs such as hSSB1 and its partner INTS3 activate ATR/CHK1 signaling (141,142).

A comprehensive study by the Lucas Lab investigated how DNA breaks occur at stalled replication forks and how ATR protects replicating DNA (143). High-throughput microscopy revealed that in the absence of ATR, RPA accumulates at sites of replication stress before DNA breakage occurs. Interestingly, stalled replication forks convert to DNA breaks in cells that have exhausted their nuclear RPA pool, leaving newly generated ssDNA uncoated and susceptible to nucleases. ATR, which is locally active at stalled replication forks, prevents unscheduled firing of dormant origins that would deplete the finite pool of RPA and induce fork breakage. It follows that depletion of the nuclear RPA pool is a catastrophic event occurring abruptly at every stalled replication fork. Hence, the abundance of RPA defines its buffering capacity for excess of ssDNA during replication stress (143). Since cancer cells often harbor high levels of intrinsic replication stress (144), these observations explain their hypersensitivity to ATR inhibitors. Along similar lines, recent work has uncovered a mechanism by which pathogens such as typhoid toxin overwhelm the RPA response to DNA damage. Evidently, typhoid toxin, through its endonuclease activity, overloads cells with ssDNA, causing RPA exhaustion that generates senescence-like phenotypes (145).

## THE MANY ROLES OF RPA IN DSB REPAIR

When the cell is challenged by genotoxic stress, damaged DNA is repaired by several pathways depending on the type of DNA damage and the cell cycle phase. Damaged bases and helix-distorting lesions in the genome are removed by base excision repair and NER, respectively, throughout the cell cycle. DSBs are repaired by DNA-PK-dependent, classical non-homologous end joining (c-NHEJ), by HDR through the pathways HR and single-strand annealing (SSA), or by alternative end joining (alt-EJ) (146).

### The role of RPA in HR

HR requires homology search and pairing of the ssDNA generated by DNA end resection with the homologous dsDNA region. DNA end resection, or simply resection, involves the nucleolytic degradation of the 5′ DNA strand that leaves long 3′ overhangs rapidly covered with RPA. Resection relies on the combined action of nucleases (MRN–CtIP, EXO1, DNA2) and helicases (BLM, WRN) (147), and RPA assists by preventing the formation of secondary structures and by shielding DNA ends from nucleolytic cleavage (148) (Figure 2B).

As a multifunctional protein, RPA not only protects ssDNA, but also regulates the activity of repair factors. RPA stimulates the activity of nucleases and helicases that carry out resection at DSBs. Biochemical evidence suggests that RPA is part of two core resection modules: BLM–DNA2–RPA–MRN and EXO1–BLM–RPA–MRN (149). It has been further demonstrated that RPA directs the 5′ → 3′ resection polarity by DNA2 while attenuating its 3′ → 5′ nuclease activity; this allows resection to occur on one strand (149–152). Binding of multiple RPA molecules to Werner syndrome protein (WRN) increases its unwinding activity and converts it into a ‘superhelicase’ (153). A recent study describes how RPA regulates EXO1-catalyzed end resection (154). The NHEJ factor KU is thought to restrict access to nucleases, such as EXO1, and to inhibit in this way resection and HDR-dependent DSB processing (155,156). Yet, an RPA–KU interaction is documented in yeast (56,73). Notably, a recent biochemical study reported a functional interplay between KU and RPA at resected DNA ends (157). In yeast, lack of KU impairs RPA and RAD51 recruitment to stalled replication forks, and attenuates HR-mediated fork restart independently of NHEJ (73). Thus, this KU–RPA interplay likely fine-tunes resection-dependent DNA repair pathways in human cells as well.

During HR repair of DSBs or stalled DNA replication forks, RPA is displaced by the RAD51 recombinase, and it is proposed that RPA, in principle, antagonizes HR by competing with RAD51 for ssDNA at DSBs (158,159). Displacement of RPA by RAD51 on ssDNA is promoted by the pro-recombinogenic mediator proteins yeast Rad52 (101,102,160,161) and human BRCA2 (162–164). Yeast Rad52 directly interacts with ssDNA-bound RPA (100,101,103), but a BRCA2–RPA interaction has not been observed (162). Recently, it has been reported that RPA–RAD51 exchange is facilitated by the small (8.3 kDa) highly acidic protein DSS1. BRCA2-associated DSS1 interacts with RPA. It is thought that the negative charges of DSS1 on its solvent-exposed acidic loop domain mimic DNA and dampen RPA’s affinity for ssDNA. As a consequence, the DSS1–RPA interaction is important for efficient HR-mediated repair in human cells (61). The RAD51-nucleoprotein filament forming after this molecular exchange promotes homology search and catalyzes strand exchange (synapsis) to drive HR. Upon strand invasion, RPA may also stabilize the displaced strand to assist recombination (165–167). Thus, HR repair is only possible during late S and G2 phases owing to the presence of the sister chromatid, which makes this repair pathway error-free.

### The role of RPA in preventing alternative error-prone DSB repair pathways

Since genetic deletion of any subunit of the RPA complex is lethal, Symington and colleagues used a heat-inducible degron system to rapidly deplete yeast RPA1 *in vivo* (148). The results show that RPA is required not only to protect the 3′ ssDNA tails from nucleolytic attack, but also to prevent annealing between short inverted repeats, which after DNA synthesis and ligation to the 5′ end can be converted to a hairpin-capped end. Moreover, extensive resection by both DNA2- and EXO1-dependent pathways is dysfunctional in the absence of RPA, as is also the recruitment of RAD51 (148). Interestingly, short ssDNA tails and low RPA levels seem sufficient to trigger checkpoint activation. Consistent with a previous report, this study suggests that a significant function of RPA is to prevent spontaneous annealing between microhomologies (148,168) (summarized in Figure 2B).

Indeed, in a follow-up study in budding yeast, the same group also dissected the requirement for resection and strand annealing during microhomology-mediated end joining, a form of KU- and ligase IV-independent but mutagenic alt-EJ (169). Using hypomorphic alleles of RPA1 to disturb the interaction between RPA and ssDNA, the authors show that the frequency of alt-EJ increases by up to 350-fold, implying that in wild-type cells spontaneous annealing between microhomologies is prevented by RPA bound to ssDNA overhangs. Furthermore, *in vitro* experiments reveal that RPA mutants are defective for ssDNA binding and the disruption of secondary structures, which allows more spontaneous annealing. Alt-EJ is frequently used to repair DSBs in mammalian cells, but has a minor role in DSB repair in budding yeast. This could be due to the presence of proteins mediating synapsis or annealing in mammalian cells, such as PARP1 and DNA ligase III, which are not present in yeast (170–173). It is thus conceivable that annealing between microhomologies is the limiting process for mutagenic alt-EJ, and that this annealing is normally suppressed by the interaction between RPA and ssDNA (169).

As mentioned earlier, annealing between interrupted inverted repeats on ssDNA results in a hairpin formation with a loop consisting of the DNA sequence between the inverted sequences. If the hairpin is located adjacent to a DSB and is left unprocessed, subsequent replication of the so formed hairpin-capped chromosome would generate inverted duplication of a palindromic sequence, and an unstable dicentric chromosome, if a centromere is present. Another study in yeast provides evidence that RPA cooperates with the nuclease activity of Mre11^MRE11^–Sae2^CtIP^ to prohibit palindromic duplications, which otherwise may lead to chromosomal rearrangements. Functional RPA antagonizes the annealing of short inverted repeats and therewith the formation of hairpins, while Mre11^MRE11^–Sae2^CtIP^ opens hairpin-capped chromosomes (174).

Considerable research has revealed that alternative error-prone DNA repair pathways in mammals are stimulated by polymerase theta (Polθ). Cancer cells defective in HR or c-NHEJ can better tolerate DNA damage through Polθ-mediated alt-EJ resulting in improved cell viability. Notably, Polθ has also been shown to negatively regulate HR (175–177). A recent study proposed that the N-terminal helicase domain of Polθ fosters the dissociation of RPA from resected DSB ends to promote ssDNA annealing and rejoining by alt-EJ. Furthermore, there is evidence that mammalian RPA promotes HR and inhibits alt-EJ of telomeric breaks *in vivo*. This study further established the function of RPA as a negative regulator of alt-EJ and described a novel antagonistic interplay between Polθ and RPA during homology-mediated DSB repair (178).

### RPA in break-induced replication

RPA was shown to play an important role during break-induced replication (BIR), a form of repair of one-ended DSBs, which also involves the formation of ssDNA intermediates. Hypomorphic mutations of yeast RPA1 that make it dysfunctional compromise RPA binding to ssDNA. Dysfunctional RPA is unable to fully protect ssDNA regions, thus compromising BIR. Notably, overexpression of RAD51 overcomes the BIR defect of RPA1 hypomorphic mutants (179).

## RPA’S POST-TRANSLATIONAL MODIFICATIONS

### Phosphorylation

**Figure 3.**
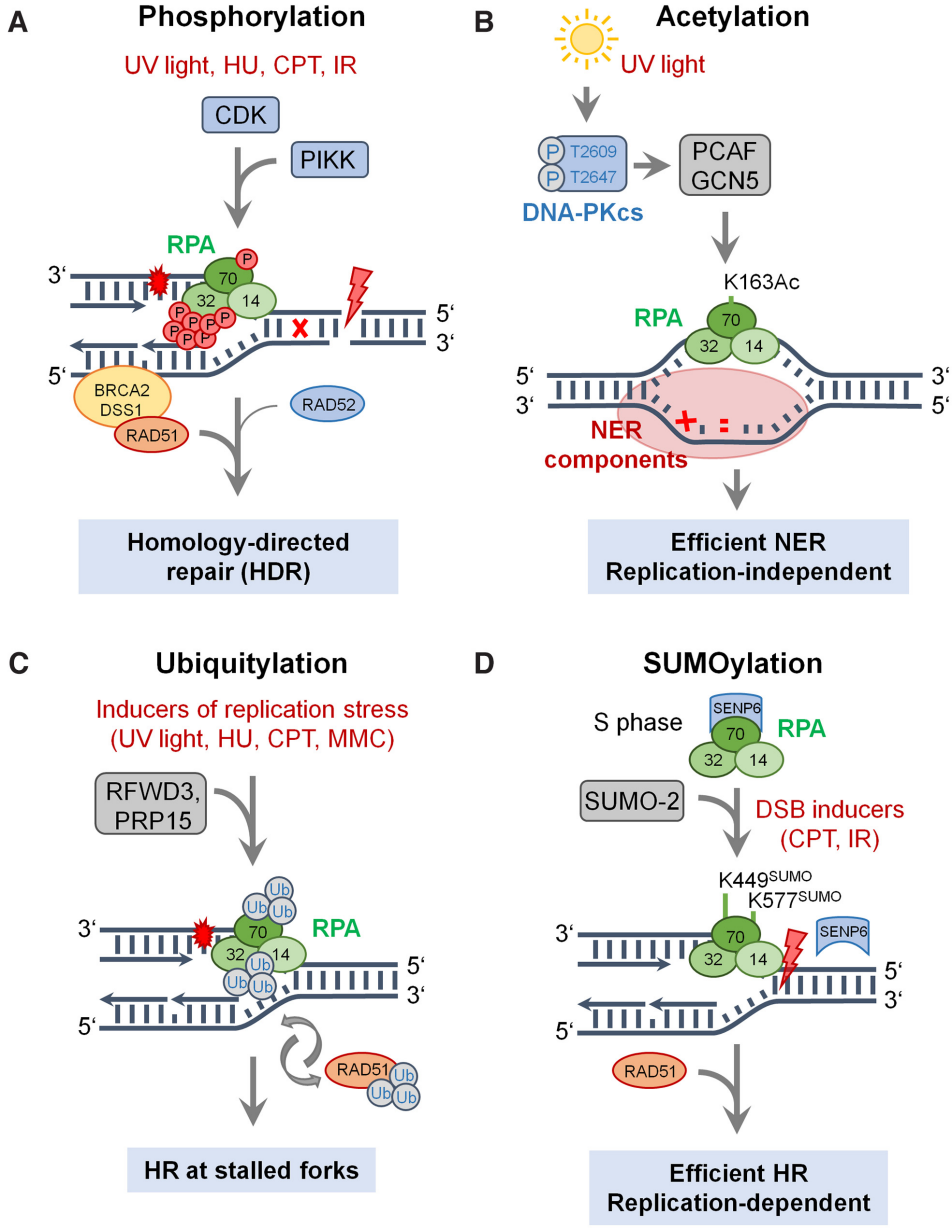
RPA modifications coordinate DNA repair. RPA’s modifications vary between the genotoxic agents and the cell cycle phase. (**A**) RPA is phosphorylated during normal cell cycle by CDKs and hyperphosphorylated at multiple serine/threonine residues by PIKK kinases in response to genotoxic stress. (**B**) UV light is the trigger for RPA acetylation primarily at a single lysine residue. (**C**) ssDNA also causes RPA ubiquitylation at multiple lysines. (**D**) Association between RPA1 and SENP6 during S phase keeps RPA70 in a hypo-SUMOylated state. Inducers of genotoxic stress are indicated in deep red. Note that other components involved in these processes are not shown for simplicity.

According to The Human Protein Atlas (180), all RPA subunits have low tissue specificity indicating broad expression across tissues. RPA protein levels do not vary significantly throughout the cell cycle but phosphorylated forms of RPA2 have been detected in S and G2 phases, while they are absent in G1 (181). Furthermore, all of RPA2 appears phosphorylated in cells blocked in mitosis, whereas only a fraction of RPA2 becomes phosphorylated in interphase cells (181). This indicates that RPA activity is regulated post-translationally. Later studies revealed that the N-terminus of RPA2 becomes phosphorylated at several Ser/Thr residues during the normal cell cycle by cyclin-dependent kinases (CDKs) (182–184), and is extensively phosphorylated in response to genotoxic stress by phosphatidylinositol 3-kinase-related kinase (PIKK) family members (60,185–194) (illustrated in Figures 1B and 3A; Table 2). Phosphorylation induces conformational changes in RPA inter-subunit interactions that may impact RPA’s interactions with many DNA repair proteins (128,195–197). The extent of RPA2 phosphorylation varies between genotoxic stress agents and cell cycle phase. It has been proposed that phosphorylation of RPA2 at S23 and S29 by CDKs stimulates S33 phosphorylation by ATR. Therefore, NBS1, a component of the MRN complex, plays an important role in RPA2-S33 phosphorylation through its direct interaction with RPA at replication-associated DSBs (198). S33 phosphorylation by ATR is critical for the subsequent and synergistic phosphorylation at other sites (T21, S12, S4 and S8) by DNA-PK and ATM (199–202). A recent study also highlights the importance of CDK-mediated phosphorylation of RPA2 in cell cycle control and DNA repair in plants (203).

**Table 2.** Overview of the RPA modifications and their proposed functions

RPA subunit and residue	Enzyme	Functional impact
*Phosphorylation*		
RPA1-T180	ATM/ATR	RPA assembly on ssDNA
RPA2-S23, -S29	CDK	Cell cycle progression
RPA2-S33	ATR	Response to genotoxic stress, DNA repair
RPA2-T21, -S12, -S4, -S8	DNA-PK/ATM	Response to genotoxic stress, DNA repair
*Acetylation*		
RPA1-K163	GCN5, PCAF	Response to UV-induced DNA damage
*Ubiquitylation*		
Multiple lysine residues on RPA1 and RPA2	RFWD3, PRP15	ATR activation, HR, interstrand cross-link repair
*SUMOylation*		
RPA1-K449, -K577	SUMO-2/3	DSB repair by HR

Great efforts have been devoted to deciphering the functional significance of RPA2’s PIKK and CDK phosphorylation sites using mutants where phosphorylatable residues are substituted by aspartate to mimic persistent phosphorylation or by alanine to create an unphosphorylatable residue (87,204–206) [reviewed in (207)]. The development of phospho-specific antibodies further strengthens these studies, which primarily focus on RPA2. Nevertheless, a study in yeast shows that RPA1 becomes also phosphorylated during checkpoint response (208), while a study in human cells maps five phosphorylation sites on RPA1 (209). Indeed, RPA1 becomes phosphorylated at threonine 180 (T180) in an ATM- and ATR-dependent manner (210). The equivalent site in yeast RPA1 (S178) is phosphorylated by the ATR homolog Mec1 during DNA replication (211). As outlined earlier, this phosphorylation event (RPA1-pS178) seems essential for the dynamic assembly of RPA on ssDNA (34).

But why is RPA2 extensively phosphorylated upon genotoxic stress? Is it a beacon? Hyperphosphorylated RPA is not associated with replication centers and therefore serves as a surrogate marker for ongoing resection at DSB sites (97,204,212). Unlimited resection of DSB ends would signal incomplete HDR and further halt progression through the cell cycle (i.e. persistent CDK1 inactivation). Additionally, hyper-resection can cause exhaustion of nuclear RPA (143). The finding that PP2A and PP4 phosphatases dephosphorylate RPA2 to complete repair by HR (213,214) is suggestive of a feedback loop between RPA phosphorylation and resection termination. A recent study described a mechanism for resection termination in eukaryotes. Normally, the physical interaction between DBD-F and BLM stimulates long-range resection. Phosphorylation of RPA changes this interaction and increases BLM’s intrinsic strand-switching activity, which slows down its DNA unwinding activity and reduces resection (215). We and others reported that error-free HR is suppressed with increasing DSB load and is counterbalanced by an increase in error-prone SSA (216–219). Since the extent of RPA2 phosphorylation depends on IR dose (i.e. DSB load) (185,194), multisite phosphorylation may serve as a threshold for inhibition of RAD51-mediated HR and a switch to RAD52-driven SSA. The massive negative charge put on RPA by phosphorylation may favor its dissociation from ssDNA, or may induce conformational changes that enhance its interaction with RAD52. Such molecular rheostats involving charge-based modifications have been observed widely in the cellular environment (220,221). In addition to phosphorylation of RPA2 on serine and threonine, evidence accumulates that multiple lysine residues are critical for additional post-translational modifications, including acetylation, ubiquitylation and SUMOylation (illustrated in Figure 3; Table 2).

### Acetylation

RPA1 is frequently identified as an acetylation target in high-throughput proteomic screenings (222,223). Thus, a small fraction of RPA1 becomes acetylated, primarily at the highly conserved lysine 163 (K163) in response to UV-induced DNA damage (123,124) (Figure 3B). NER is the main DNA repair mechanism that removes bulky DNA lesions induced by UV light and environmental mutagens, and the involvement of RPA in NER is well documented (14,120,224–226). This modification at RPA1-K163 is mediated by the combined action of the acetyltransferases GCN5 and PCAF and serves to enhance the interaction between RPA1 and XPA. Thus, retention of this crucial component of NER is achieved at the UV damage sites. Suppression of RPA1 acetylation causes hypersensitivity to UV irradiation by compromising the removal of cyclobutane pyrimidine dimers and 6–4 pyrimidine–pyrimidine photoproducts. Interestingly, DNA-PK is the main upstream kinase required for UV-induced RPA1 acetylation, and chemical inhibition of its activity dramatically reduces RPA1 acetylation. K163 acetylation of RPA1 is reversed by HDAC6 and SIRT1 deacetylases (123,124). In yeast, NuA4 histone acetyltransferase complex is recruited to resected DNA by MRX and causes RPA acetylation. Notably, the Nu4A–RPA interaction is DNA damage dependent and causes the displacement of RPA from ssDNA (227).

### Ubiquitylation

Several proteomic studies (228,229) also report the ubiquitylation of RPA1 and RPA2. Thus, the E3 ubiquitin ligase RING finger and WD repeat domain 3 (RFWD3) is recruited to DNA damage sites and physically associates with RPA (104,105) (Figure 3C). Elledge’s lab has further shown that the entire chromatin-bound fraction of RPA is indeed multiply ubiquitylated after UV treatment (230). RPA ubiquitylation mediated by RFWD3 does not trigger proteasomal degradation, but serves instead to promote HR at stalled replication forks (230). Recent reports show RFWD3-mediated polyubiquitylation of both RPA and RAD51 in response to mitomycin C-induced damage, to facilitate their clearance from the damage sites (and thus HR completion) by the ubiquitin-selective segregase VCP/p97 and the proteasome (231). VCP/p97 has previously been implicated in the regulation of DDR by removing chromatin-bound proteins (232,233). Mutations in RFWD3 or RPA2 that disrupt the RFWD3–RPA interaction are also associated with defects in interstrand cross-link repair (234).

Further work by Zou’s lab identified another E3 ubiquitin ligase, PRP19, that acts as a sensor for RPA–ssDNA via its interaction with RPA (91). PRP19 is a well-known regulator of pre-mRNA splicing, but can independently also ubiquitylate RPA2 with K63-linked chains in response to DNA damage or replication stress, thus promoting ATRIP recruitment. Thus, PRP19 is not involved in protein degradation, but instead reinforces the full activation of ATR on RPA–ssDNA and the associated downstream events (91). Collectively, these studies establish RPA–ssDNA as a platform for ubiquitylation during DDR that shows similarities to the γH2AX ubiquitylation platform via the ubiquitin ligases RNF8 and RNF168 (91,235,236). Finally, the E3 ubiquitin ligase HERC2 is also implicated in RPA2 ubiquitylation, but the mechanistic significance of this modification remains to be elucidated (69).

### SUMOylation

Mammalian RPA1 undergoes SUMOylation at lysine residues K449 and K577 (Figure 3D). The SUMO-specific protease SENP6 keeps RPA1 in a hypo-SUMOylated state during normal DNA replication. However, induction of DSBs, either by CPT or by IR, triggers the dissociation of RPA1 and SENP6, which then allows RPA1 SUMOylation by SUMO-2/3. SUMOylation of RPA1 enhances its interaction with RAD51 and promotes HR (107). The yeast homolog of RPA also undergoes SUMOylation, often at multiple sites, after DNA damage (237), and SUMOylated RPA1 contributes to checkpoint activation by enhancing interaction with Sgs1/BLM helicase (238). It should be noted, however, that the predicted SUMOylation sites in mammalian RPA1 are not conserved in yeast RPA1. RPA is not only a target for SUMOylation, but also required for SUMOylation of Rad52 and Rad59 HR factors in budding yeast through the interaction of RPA2 with SUMO ligase Siz2 (239). These findings demonstrate that distinct RPA modifications have the potential to modulate DNA repair pathway choice.

## RPA IN OTHER ASPECTS OF DNA METABOLISM

### A role for RPA in cGAS-STING pathway

Newer reports document the engagement of RPA in DNA transactions other than those in DNA replication and repair described earlier. Exogenous nucleic acids such as microbial and viral DNA from infectious agents, as well as siRNA and miRNA, can trigger inflammatory responses activating type I interferon (IFN). Moreover, DNA repair involves the excision of short ssDNA by-products, which in mammalian cells are cleared by the cytosolic nuclease TREX1. Therefore, TREX1 deficiency results in the accumulation of self-DNA in the cytoplasm that initiates inflammatory responses causing autoimmune disease (240,241). In mammalian cells, cytosolic nucleic acids are sensed by cGAS-STING and RIG-I/MDA5 pathways, which detect cytosolic DNA and dsRNA, respectively (242,243). However, these receptors have limited ability to distinguish between self *and* non-self nucleic acids, which suggests the existence of additional mechanisms. Indeed, a cell intrinsic mechanism for nuclear retention of ssDNA has been described involving the ssDNA-binding capacity of RPA and RAD51. Depletion of RPA and RAD51 leads to leakage of ssDNA into the cytosol and type I IFN activation in a cGAS-dependent manner. Although TREX1 is not directly involved in DNA repair due to its cytoplasmic localization, TREX1 deficiency increases the levels of ssDNA in the cell nucleus and can thus cause RPA and RAD51 exhaustion, which in turn causes accumulation of ssDNA in the cytosol (244).

### RPA in retrotransposition

Long interspersed elements (LINEs) are autonomously active retrotransposons that can move to new locations in a genome by reverse transcription. LINEs are 6–8 kb in length and comprise ∼21% of the human genome (245). As such, LINEs can disturb genome integrity during early embryonic development through insertions, deletions or rearrangements, thus contributing to genomic variation but also causing novel diseases (246). Therefore, cells have evolved mechanisms to combat retrotransposition (247). Notably, proteins involved in DNA replication and/or repair can impact retrotransposition (248–253). It has been reported that poly(ADP-ribose) (PAR) polymerase 2 (PARP2) is recruited to and activated by ssDNA breaks generated at LINE-1 (or L1) endonuclease cleavage sites to generate PAR chains, which structurally resemble single-stranded RNA or DNA. This triggers the recruitment of RPA at L1 integration sites to facilitate retrotransposition. Interestingly, RPA can also guide the cytidine deaminase APOBEC3A to sites of L1 integration (254) that can generate a cytosine to thymine mutation. This is reminiscent of previous studies, which reported that RPA can interact with the AID to mediate somatic hypermutation and class switch recombination of immunoglobulin genes (46,255). Although APOBEC is part of the immune defense functioning by restricting retroviruses and the mobility of endogenous retroelements (256), it is also possible that RPA protects ssDNA L1 integration intermediates from cytidine deamination by APOBEC3A (254). This is in agreement with a study in yeast, demonstrating that RPA limits the processing activity of editing deaminases on ssDNA (257).

## RPA IN THE TRANSCRIPTION WORLD AND IN RNA METABOLISM

Cell division requires genome-wide transcriptional changes. There are three main transcriptional waves accompanying the different transition points during the cell cycle—G1-to-S, S-to-G2 and G2-to-M (258). During transcription, R-loop structures can naturally form, where the RNA transcript transiently pairs with the coding DNA strand to form a DNA–RNA hybrid, leaving the non-coding DNA single-stranded and thus accessible to SSBs for shielding. The displaced ssDNA in R-loops is likely to be recognized by RPA in the absence of DNA damage (Figure 2C). Although R-loops emerge as potential regulators during transcription and DNA repair, they can also negatively affect genome integrity under certain conditions. Thus, while short DNA–RNA hybrids are naturally transiently formed during transcription, persistent re-annealing of the transcript RNA to the template DNA strand can impair transcription and trigger the DDR. Activation of DDR also occurs when impaired removal of RNA primers during lagging-strand DNA synthesis results in replication stress (259,260).

In an effort to identify promoter-bound pre-initiation complexes (PICs) using a quantitative proteomic screen in budding yeast, RPA1 and another ssDNA-binding protein, Sub1, were found to associate with RNA polymerase II (RNAPII) complex (261). In contrast to Sub1, which is recruited predominantly to transcription start sites, RPA1 is excluded from promoter and intergenic regions, but is localized downstream of promoters in transcribed regions of active genes, independently of ongoing replication. Additionally, ChIP analysis reveals that RPA1 is also present at genes transcribed by RNAPIII. Given the observed synthetic genetic interactions between RPA1 mutants and the elongation factors Spt4 and Bur2, this study suggests a role for RPA in transcription elongation (261), extending previous reports that link RPA to transcription regulation (262,263). The authors propose that RPA binds the non-template strand during transcription elongation, while Sub1 binds predominantly at the transcription bubble, where the two DNA strands are dissociated. An intriguing possibility is that in this way RPA prevents strand invasion of resected ssDNA to suppress unwanted recombination (261).

Turning to transcription-associated DSBs, an impressive study in fission yeast using I-Ppol-induced DSBs at rDNA repeats reports that loss of RNaseH, the ribonuclease degrading RNA in DNA–RNA hybrids, stabilizes such hybrids around DSBs and prevents RPA recruitment. RNaseH overexpression has the opposite effect: unstable DNA–RNA hybrids associated with enhanced resection and recruitment of RPA (264). Indeed, it has been demonstrated that in this yeast RNaseH is necessary for efficient HR and the recruitment of RNAPII at I-Ppol-induced DSB sites. Notably, RNAPII can initiate transcription at the 3′ ssDNA overhangs without the assembly of PIC resulting in the formation of DNA–RNA hybrids. Since these hybrids counteract the recruitment of RPA, RNaseH activity is necessary to eliminate the RNA moiety and ensure full RPA loading and completion of the repair process. Intriguingly, RNaseH overexpression correlates with loss of repeat regions when DSBs occur in repetitive regions, for example rDNA repeats. It has been concluded that DNA–RNA hybrids exert a protective role during HR repair against unwanted intrachromosomal recombination between repeat regions (264). The findings in this extensive study may overturn the long-held model of HR. A later study in mammalian cells has demonstrated that DNA–RNA hybrids form predominantly during S/G2 phases and downstream of end resection. These DNA–RNA hybrids are formed by annealing between the resected DSB ends and the damage-induced long non-coding RNAs transcribed from the broken ends. Loss of RNaseH, however, does not affect end resection and RPA foci formation. Furthermore, proximity ligation assays reveal an interaction between RNaseH2A subunit and RPA upon IR-induced DNA damage (265). The presence of both RPA and RNaseH1 at R-loops in human cells is also detectable by immunofluorescence and ChIP (106). An *in vitro* assay with an R-loop substrate revealed that RPA directly promotes the activity of human RNaseH1, but not *E. coli* RNaseH1 (106). Similarly, *E. coli* RNaseH1 directly interacts with the *E. coli* ortholog of the eukaryotic RPA complex (266). These findings suggest that the regulation of RNaseH1 by SSBs is evolutionarily conserved and has an important role in suppressing of R-loop-associated DNA damage. In addition to RNaseH, the RNA exosome is also able to remove *de novo* transcribed RNA at defined DSB sites to enable RPA recruitment and efficient HR repair (267,268).

Notably, a recent biochemical study demonstrates that similar to ssDNA, RPA is also able to bind ssRNA in a highly dynamic manner, albeit with weaker affinity. Thus, although RPA binds ssDNA of 10, 20 or 30 nt length, it only binds RNA of 30 nt or longer (269). In contrast, SSBs of the hyperthermophilic *Saccharolobus solfataricus* bind ssRNA as efficiently as ssDNA and protect it from degradation by the archaeal exosome (270). SSBs from other thermophilic species also bind viral RNA efficiently and likely modulate viral RNA metabolism (271). From an evolutionary perspective, temperature decrease may account for more specialized functions of ubiquitous proteins binding to single-stranded nucleic acids.

Apart from its ssDNA-binding activity, RPA is also implicated in gene expression through interactions with transcription factors. One such transcription factor is the tumor suppressor p53, which forms a complex with RPA (81–83) and suppresses HR (84). Indeed, it has been reported that the DNA-PK/ATM/ATR kinase module affects p53–RPA interactions during HR, with DNA-PK phosphorylating RPA2 and ATM/ATR phosphorylating p53. Simultaneous phosphorylation of both p53 and RPA enables their dissociation causing the release of active p53 and promoting HR (85).

RPA is also involved in the transcriptional regulation of human metallothionein (262) and the endothelial nitric oxide synthase (272). RPA1 is required for the transcriptional activation of BRCA1 (273) and heat shock factor 1 target genes (66). The latter occurs by recruiting the histone chaperone FACT, which displaces histones and opens up chromatin (66). Recently, it has been reported that RPA1 binding to the transcription factor NRF2 is involved in the suppression of *MYLK* transcription (274). Notably, RPA physically interacts with the histone chaperone HIRA at gene promoters and enhancers. In the proposed model, RPA recruits HIRA to gene regulatory elements and regulates HIRA-mediated deposition of newly synthesized histone H3 variant, H3.3 (71). These studies in aggregate demonstrate that RPA not only functions as the major ssDNA-binding protein in human cells, but is also involved in fine-tuning the regulation of gene expression.

## RPA AT TELOMERES

Telomeres are regions with repetitive DNA sequences at the ends of linear chromosomes that terminate in ssDNA overhangs comprised of G-rich 3′ ends. These natural ends of linear chromosomes resemble DSBs with resected ends. To prevent unwanted ‘repair’ that would lead to chromosomal end-to-end fusions, telomeres are protected from recognition by the DNA repair machinery by a specialized shelterin complex, as well as by a lariat structure known as telomere loop that hides the DNA end. Moreover, G-rich telomeric DNA repeats can fold spontaneously into G-quadruplexes (G4s). G4 formation at telomeric overhangs impedes telomerase activity, a ribonucleoprotein complex responsible for maintaining telomere length through reverse transcription.

Since telomeres contain ssDNA regions, it is not surprising that RPA is naturally involved in telomere biology (275–278). Human RPA efficiently unfolds telomeric G4 structures *in vitro* (279–285). Moreover, the function of the fission yeast Pif1 helicase in unwinding G4 structures depends on RPA and positively regulates telomere length (286). RPA and mtSSB also collaborate with Pif1 helicase in melting G4 structures during mitochondrial DNA replication (287). In fission yeast, the RPA1-D223Y mutation causes severe replication defects at telomeres, accumulation of G4 structures and increased recruitment of HR factor Rad52, while overexpression of Pif1 overcomes these defects (288).

Protection of telomeres 1 (POT1), a protein interacting with telomeric ssDNA, is also implicated in G4 unwinding *in vitro* (289). It is likely that during replication of the lagging telomere strand, RPA is recruited at telomeres by the replication machinery. After DNA synthesis, RPA is displaced by POT1, in a process mediated by telomeric repeat-containing RNA (TERRA) and heterogeneous nuclear ribonucleoprotein A1 (hnRNPA1) (290,291). It is therefore likely that G4 formation at telomeres and POT1 loading suppress DNA damage signals mediated by RPA (292).

Notably, a telomere-specific RPA-like heterotrimeric complex, CST (Cdc13–Stn1–Ten1), protects telomeres independently of POT1 (293,294). RPA suppresses *in vitro* resection at telomeres in collaboration with Cdc13, the main component of the CST complex, suggesting an interplay between these two ssDNA-binding complexes (295). RPA also facilitates the activity of telomerase in late S phase in budding and fission yeast as part of a transient complex comprising RPA, Ku, Cdc13 and telomerase (56). Interestingly, shared subunits of RPA complex and telomerase holoenzyme have been reported in the ciliate *Tetrahymena thermophila*. These RPA-like complexes have distinct functions in different cellular contexts (296).

## RPA HOMOLOGS AND EQUIVALENTS

A human homolog of RPA2, named RPA4, was identified that associates with RPA1 and RPA3 to form an alternative complex (aRPA), which efficiently binds ssDNA (297), but is unable to support DNA synthesis leading to cell cycle arrest (298,299). Notably, RPA4 is preferentially expressed in non-proliferating, quiescent cells and supports DNA repair and thus genome maintenance (297,300,301).

HR is fundamental to the maintenance of genetic diversity during meiotic crossover events. Owing to embryonic lethality of RPA1–3 mutant mice, the role of RPA in meiotic recombination is less well known (302). A recent study using an inducible germline-specific *RPA1* deletion approach demonstrates that RPA is essential for meiotic recombination in mice (303). In addition, meiosis-specific with OB domain (MEIOB) is a recently discovered meiosis-specific RPA1 homolog in metazoans (304,305). MEIOB contains OB-fold domains, homologous to those of RPA1, but lacks its conserved N-terminal protein interaction domain (304,305). Moreover, MEIOB exhibits ssDNA-specific 3′-exonuclease activity that explains why RPA1 cannot compensate for the absence of MEIOB in mice (304,305). MEIOB can form a complex with RPA2 and the meiosis-specific protein SPATA22 (305). However, multiple combinations of MEIOB, SPATA22 and the different RPA subunits are also possible (306).

In addition to the above RPA homologs, two additional human SSB proteins have been identified, hSSB1 and hSSB2, that are more closely related to bacterial and archaeal SSBs than to RPA (307,308). Each of these homologs is a component of a heterotrimeric complex, sensor of ssDNA (SOSS), together with SOSS-A (INTS3) and SOSS-C (C9orf80), and exerts important functions in the cellular responses to DNA damage and the maintenance of genomic stability (309–311).

Mitochondria contain their own SSB proteins involved in mitochondrial DNA replication and maintenance. Human mtSSB (HmtSSB) binds to ssDNA as homo-tetramer, comprised of four identical ∼16 kDa subunits (312,313). HmtSSB tetramer binds to ssDNA in two distinct binding modes depending on the length of ssDNA (30 and 60 nt), salt concentration and the gradual generation of ssDNA (314,315). HmtSSB is structurally similar to *E. coli* SSB (EcoSSB) but lacks the disordered C-terminal domain present in EcoSSB (313). Nevertheless, both proteins share common physicochemical properties (316). Rim1 is the mtSSB in budding yeast (317), which was shown to form unstable tetramers in solution (318). It has been postulated that Rim1 binds to ssDNA as a dimer, followed by binding of a second one to form tetramers on DNA (318).

## TARGETING RPA FOR EFFECTIVE CANCER THERAPY

**Figure 4.**
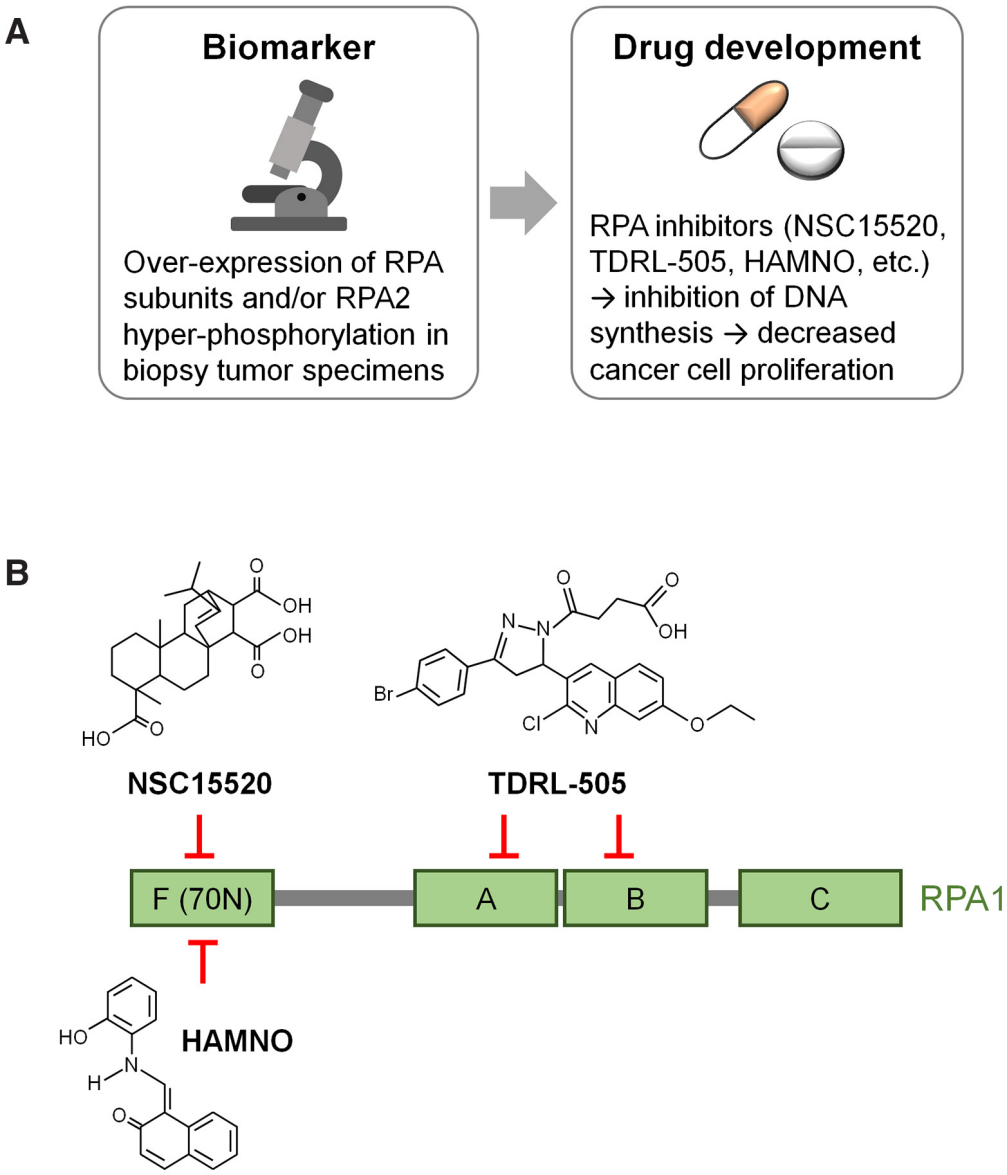
Clinical applications of RPA. (**A**) Overexpression of RPA subunits or hyperphosphorylation of RPA2 may serve as a prognostic biomarker in tumor specimens. This fact guided the development of novel RPA inhibitors, which have the potential to inhibit DNA replication in cancer cells. (**B**) Examples of well-characterized small molecule inhibitors targeting RPA1 subunit. NSC15520 (fumaropimaric acid) and HAMNO ((1*Z*)-1-[(2-hydroxyanilino)methylidene]naphthalen-2-one) target the N-terminal DBD-F domain, which is involved in protein–protein interactions. TDRL-505 targets the central ssDNA-binding domains of RPA1, DBD-A and DBD-B.

Cancer is a condition of uncontrolled cell proliferation, and DNA replication stress has been linked to the progression of this disease (137,144,319). Therefore, one way to effectively treat cancer could be through targeting the replication stress response. Since RPA is the major SSB protein that is essential for DNA synthesis, activity inhibition or downregulation would put a break on cancer cell proliferation (Figure 4A). Several studies report promising results in this direction and are reviewed next.

In mice, a heterozygous missense mutation in *RPA1* (L230P) leads to the development of lymphoid tumors (320). Biallelic somatic mutation of *RPA1* has been found in a pancreatic tumor (321). Overexpressed RPA1 and/or RPA2 are detected in various cancers, suggesting that RPA may be useful as a prognostic marker in cancer patients (322–330). Elevated RPA3 expression is also implicated in the development of gastric (330,331) and hepatocellular carcinoma (332). The oncogenic properties of RPA appear linked to the cyclin D pathway (322,325,329), which drives the G1/S-phase transition. It is therefore relevant that overexpression of microRNA 30a slows down proliferation of ovarian and gastric cancer cell lines by targeting RPA1. This hampers replication, causes DNA fragmentation, activates the S-phase checkpoint and induces p53-mediated apoptotic cell death (333).

Screening for compounds inhibiting proliferation of non-small cell lung cancer (NSCLC) cells uncovered ailanthone, a natural compound with herbicidal activity isolated from *Ailanthus altissima*, as a promising candidate (334). Gene expression analysis revealed that ailanthone exerts its antiproliferative effect by mainly downregulating the expression of RPA1, at both the mRNA and the protein level. This inhibition of RPA function suppresses DNA replication and NSCLC cell proliferation *in vitro* and growth of tumor xenografts and of orthotopic tumor models *in vivo* (334). However, the effect of the compound on the proliferation of non-cancerous cells remains to be investigated.

RPA also interacts with tumor suppressor genes like menin, a protein regulating NF-κB transactivation, frequently lost in multiple endocrine neoplasia type 1 (74,335). Of note, RPA2 overexpression is implicated in the general pathogenesis of cancer (335) and its ectopic expression in breast cancer cells abrogates menin/NF-κB–p65 complex formation and unleashes the expression of NF-κB-regulated oncogenes (75). Therefore, targeting of RPA2–menin interaction in breast cancer cells may be a promising therapeutic strategy.

Other studies report tumor suppression mechanisms that involve the regulation of RPA1 during DNA replication by PTEN (92). PTEN functions as a tumor suppressor that localizes to replication sites and physically interacts with the RPA1 C-terminus. PTEN promotes RPA1 protein stability by regulating its ubiquitylation status, most likely by recruiting the deubiquitinase OTUB1, thus protecting stalled replication forks (92).

S4/S8-RPA2 phosphorylation appears to be a useful indicator of cancer progression in oral squamous cell carcinomas (336). Notably, a significant increase in S4/S8-RPA2 phosphorylation, suggesting DDR activation, has been observed in dysplastic tissues, which gradually declines as the tumor progresses to later stages (336). This observation is in line with the model that DDR acts as an early barrier to tumorigenesis (337,338). Disruption of RPA phosphorylation may be another way to attack cancer cells. Thus, valproic acid, a histone deacetylase inhibitor, and hydroxyurea, a ribonucleotide reductase inhibitor, synergistically sensitize breast cancer cells by perturbing RPA2 hyperphosphorylation and thus HR (339). Finally, since RPA2 is extensively phosphorylated in cancer cells with high levels of replication stress and abrogated CHK1, it can be used as a predictive biomarker in cancer therapy protocols utilizing CHK1 inhibitors (340).

RPA exhaustion induced by high levels of replication stress and NER deficiency promotes sensitivity to cisplatin in ovarian cancer cells, possibly by MRE11-mediated degradation of nascent ssDNA at stalled forks, and can be used as a strategy to treat cancer. Conversely, ectopic overexpression of RPA subunits overcomes these effects (341). Thus, modulating RPA availability may be a useful strategy particularly when drug resistance occurs. Similarly, downregulation of RPA affects RAD51 recruitment to DSBs and enhances the radiosensitivity of nasopharyngeal cancer cells (342). These studies in aggregate provide an explanation as to why overexpression of RPA in various cancers is predictive for unfavorable outcome (322–332).

An alternative, PIKK-independent regulatory module for HR has been reported in cancer cells. It involves the phosphorylation by casein kinase 2 of the histone methyltransferase G9a and its recruitment to chromatin. G9a interacts with RPA and promotes RPA–RAD51 exchange at DSBs, thus promoting HR and cell survival (343). A correlation between G9a and RPA-mediated DDR has been observed in colon cancer stem cells (344). Hence, combination of RPA and G9a inhibitors is expected to have synergistic effects on cancer cell death. All these studies advocate the potential of RPA as a therapeutic target and the need to find effective RPA inhibitors.

A way to modulate RPA–protein interactions in cancer cells, and thereby to disrupt DDR activation, is via specific inhibitors that target the N-terminus or RPA1 (70N) and the C-terminus of RPA2 (32C), which harbor the protein interaction modules (Figure 1). Several small molecules inhibiting the ssDNA-binding activity of RPA have been reported (Figure 4B). TDRL-505 is cytotoxic both as a single agent and in combination with other chemotherapeutics (345). Its isobornyl derivatives MCI13E and MCI13F induce apoptosis in lung and ovarian cancer models and show synergy with cisplatin in combination treatment protocols (346,347). Another RPA inhibitor, NSC15520, does not prevent binding of RPA to ssDNA, but disrupts DBD-F interactions with p53 and Rad9, possibly affecting in this way downstream genome integrity pathways (348,349).

HAMNO, a further RPA inhibitor, also targets the N-terminal domain of RPA1. HAMNO prevents the autophosphorylation of ATR and ATR-mediated phosphorylation of RPA2 at S33. Consequently, HAMNO elevates DNA replication stress and mitigates tumor growth (350). A recent report demonstrates that HAMNO sensitizes glioblastoma cancer stem-like cells to ionizing radiation (351). The potential of other RPA inhibitors (352–356) as cancer therapeutics or as chemosensitizing agents needs to be validated. Moreover, an important aspect to consider is that the effect of RPA inhibitors on cancer treatment may not only arise from replication stress. Since RPA suppresses error-prone processes like alt-EJ and cytosine deamination, inhibiting RPA would potentiate genome instability and cell death.

The inhibitors discussed above function by preventing RPA interaction with ssDNA and/or repair proteins. An additional strategy for inhibition of RPA function is by reducing its mobility via chemical cross-linking. UV light is frequently used as a cross-linking agent to immobilize biomolecules. However, solar UV irradiation is genotoxic to the skin and contributes to the development of skin cancer. UV-induced oxidative damage is not restricted to nucleic acids and there is evidence that it also affects RPA. Reports show that oxidatively damaged RPA compromises NER, owing to UV-induced covalent cross-linking between RPA1–3 subunits that limits RPA conformational changes when bound to ssDNA (357).

## NEW TOOLS TO STUDY RPA DYNAMICS ON DNA AND THEIR APPLICATIONS IN DIAGNOSTICS

The dynamic binding of RPA on the ssDNA substrate and the binding between RPA and RAD51 are of immense interest. ‘DNA curtains’ is a technique developed in Greene’s lab for single-molecule fluorescence imaging of protein–nucleic acid interactions, including RPA binding to ssDNA in the presence of multiple DNA-binding proteins (98,215,358–361). Briefly, ssDNA is synthesized by rolling circle replication, biotinylated at one end and anchored on a lipid bilayer. Application of hydrodynamic force aligns the DNA in the direction of flow. Introduction of fluorescently tagged (e.g. by GFP) SSBs allows labeling of the DNA and the elimination of secondary structures (362).

To monitor RPA dynamics on ssDNA in a multiprotein reaction, a fluorescently labeled version of yeast RPA (RPA^f^) was engineered by incorporating a chemical fluorophore into RPA2 using non-canonical amino acids and bio-orthogonal chemistry. Upon binding to ssDNA, RPA^f^ undergoes a change in fluorescence that can be quantified. This approach circumvents the drawbacks of large-protein fusions, which may affect protein behavior, *in vitro* or in the complex cellular environment. This approach to RPA labeling with fluorophores enables investigation of RPA dynamics in multiple DNA processes (30).

An alternative approach utilizes a nuclease-deficient CRISPR–Cas9 system to induce ssDNA regions at human telomeres. Localization of nuclease-deficient Cas9 to telomeres with a single-guide RNA complementary to telomeric repeat DNA leads to the formation of RNA–DNA duplexes that leave one telomeric DNA single-stranded and capable of recruiting RPA and other factors involved in DDR. This model can be used to study RPA recruitment in G1 cells and has potential for application on other genomic repeats (363).

The DBD-A of human RPA1 has also been employed in a very creative way to improve the detection of disease biomarkers. RPA1 conjugated with gold nanoparticles (AuNPs) can be used to increase the sensitivity of paper-based lateral flow immunoassays, which normally allow only a limited number of antibody-conjugated AuNPs to bind the target protein. Since RPA binds ssDNA in a sequence-independent manner, the antibody is replaced by an aptamer (short oligonucleotides) against the target. Signal enhancement is achieved by the attachment of several RPA1-conjugated AuNPs per aptamer. Using this approach, the influenza virus nucleoprotein and the cardiac troponin I could be detected, paving the way to the detection of other biomarkers requiring higher sensitivity (364).

Another example of an on-site sensitive diagnostic tool based on aptamer–RPA1A interaction is the colorimetric detection of nucleocapsid protein (NP) of severe fever of thrombocytopenia syndrome virus (SFTSV). In this case, RPA1A is conjugated to the surface of liposomes with enzyme encapsulation, while a novel aptamer specific for SFTSV NP is bound to a pre-fixed antibody. The interaction between RPA1A on the surface of the liposome and the aptamers enables target detection in a colorimetric reaction following liposome lysis (365).

## CONCLUSIONS

In summary, our mechanistic understanding of how RPA functions in eukaryotic DNA synthesis and repair and the associated checkpoint control is getting broader. New functions of RPA emerge, as it appears involved in all nucleic acid transactions, where ssDNA is transiently generated. Advances on the role of RPA in cancer and the potential of development of specific small molecule inhibitors open new avenues in cancer prevention and treatment. Finally, the RPA’s ssDNA-binding properties offer unique opportunities for the development of novel diagnostic tests. Certainly, a lot more excitement should be expected from RPA in the coming years.
